# Radiolabeled iron oxide nanoparticles functionalized with PSMA/BN ligands for dual-targeting of prostate cancer

**DOI:** 10.3389/fnume.2023.1184309

**Published:** 2023-09-20

**Authors:** Danae Efremia Bajwa, Evangelia-Alexandra Salvanou, Maria Theodosiou, Theodora S. Koutsikou, Eleni K. Efthimiadou, Penelope Bouziotis, Christos Liolios

**Affiliations:** ^1^ Radiochemical Studies Laboratory, Energy & Safety, Institute of Nuclear & Radiological Sciences & Technology (INRASTES), National Centre for Scientific Research (NCSR) “Demokritos”, Athens, Greece; ^2^ Laboratory of Inorganic Chemistry, Department of Chemistry, National and Kapodistrian University of Athens, Athens, Greece; ^3^ Research Laboratory, Institute of Pharmaceutical Research & Technology (IFET) (Pallini), Athens, Greece

**Keywords:** prostate cancer, PSMA, bombesin, bispecific heterodimers, iron oxide nanoparticles, ^99m^Tc, SPECT

## Abstract

**Introduction:**

Prostate cancer (PCa) is the second most frequent cancer diagnosis in men and the fifth leading cause of death worldwide. Prostate Specific Membrane Antigen (PSMA) and Gastrin Releasing Peptide (GRP) receptors are overexpressed in PCa. In this study, we have developed iron oxide nanoparticles (IONs) functionalized with the Prostate Specific Membrane Antigen (PSMA) and Gastrin Releasing Peptide (GRP) ligands for dual targeting of Prostate cancer.

**Methods:**

IONs were developed with a thin silica layer on their surface with MPTES (carrying -SH groups, IONs-SH), and they were coupled either with a pharmacophore targeting PSMA (IONs-PSMA) or with bombesin peptide (IONs-BN), targeting GRP receptors, or with both (IONs-PSMA/BN). The functionalized IONs were characterized for their size, zeta potential, and efficiency of functionalization using dynamic light scattering (DLS) and Fourier-Transform Infrared Spectroscopy (FT-IR). All the aforementioned types of IONs were radiolabeled directly with Technetium-99m (^99m^Tc) and evaluated for their radiolabeling efficiency, stability, and binding ability on two different PCa cell lines (PC3 and LNCaP).

**Results and Discussion:**

The MTT assay demonstrated low toxicity of the IONs against PC3 and LNCaP cells, while the performed wound-healing assay further proved that these nanostructures did not affect cellular growth mechanisms. The observed hemolysis ratio after co-incubation with red blood cells was extremely low. Furthermore, the ^99m^Tc-radiolabeled IONs showed good stability in human serum, DTPA, and histidine, and high specific binding rates in cancer cells, supporting their future utilization as potential diagnostic tools for PCa with Single Photon Emission Computed Tomography (SPECT) imaging.

## Introduction

Prostate cancer (PCa) is the most frequent cancer diagnosed in men and the second most fatal cancer in the same category of patients, while being the fifth leading cause of death worldwide (1). The prostate-specific antigen (PSA) test, which became available in the late 1980s, offers a simple and affordable way to find asymptomatic men, who may be harboring an earlier stage of prostate cancer, thus increasing the likelihood of cure, and ultimately lowering prostate cancer-specific mortality (2, 3). Recent advancements in imaging technology, particularly hybrid combinations of Magnetic Resonance Imaging (MRI) and Single-Photon Emission Computerized Tomography (SPECT), may significantly improve lesion detection and staging, especially for local staging, diagnosis, therapy and follow-up of prostate cancer (4, 5). While MRI generates pictures with high spatial resolution but of low sensitivity, SPECT is quantitative and has high sensitivity but poor spatial resolution. Therefore, hybrid technologies, such as SPECT/MRI, enable further advancement in molecular imaging, by combining the strong points of each imaging technology, i.e., the sensitivity of molecular targeting with anatomic specificity. SPECT/MRI has received limited focus in pre-clinical research, despite its potential to provide a nanomolecular diagnosis with valuable information for the severity and distribution of the disorder (6), especially if suitable imaging probes are being developed, i.e., magnetic nanoparticles labeled with SPECT radionuclides.

The use of nanoparticles as drug carriers could be beneficial in the treatment of cancer. Due to their enhanced selectivity, these nanocarriers significantly boost the effectiveness of the medicine they carry, while minimizing adverse effects on the host (7, 8). Functionalization with targeting molecules that are specific to cell organelles can increase the efficacy of the administered pharmaceuticals even further (9). Tissues, organs, and individual cells can all be targeted using nanoscale platforms, which exist in a variety of materials, geometries, sizes, and targeting moieties (10). They have a low cost for their manufacturing, sustained and regulated release characteristics, scale-up capability, and no immunogenicity (9). Due to these favorable properties, nanoparticles have also been proposed as viable delivery systems for various imaging agents for concurrent disease monitoring using multiple modalities (11).

Pure metals, metal alloys, and metal oxides comprise magnetic nanoparticles (MNPs). Iron oxide nanoparticles (IONs) are among the most desired nanostructures in nanomedical sciences because of their exceptional physicochemical properties (superparamagnetic), low levels of toxicity, stability in aqueous solutions, and biocompatibility. Their low oxidation sensitivity is what causes the magnetic response to be consistent (12). These nanoparticles can be used for radiotherapy, hyperthermia, and cancer diagnosis using MRI and/or Positron Emission Tomography (PET) nuclear imaging, SPECT, and x-ray computed tomography. They can also be used as targeted drug delivery systems using an external magnetic field or by attaching a suitable targeting group to the surface of the nanoparticles (13).

The type II transmembrane protein PSMA consists of a 707-amino-acid extracellular section that hydrolyzes N-acetyl-L-aspartyl-L-glutamate (NAAG) to N-acetylaspartate (NAA) and L-glutamate and a 19-amino-acid intracellular portion, while the PSMA gene (FOLH1) is located on the short arm of chromosome 11 (14). PSMA is present in normal human prostate cells (15), while in PCa cells it is over-expressed as a membrane protein especially in high-grade and metastatic PCa (16, 17). As a result, PSMA has become one of the best candidates for PET imaging of PCa, and recently peptidomimetic PSMA inhibitors based on the scaffolds: Glu-CO-Glu-OH and Lys-CO-Glu have demonstrated outstanding performance as radioligands for PET diagnostic imaging (^68^Ga-PSMA-11) and PCa treatment (^177^Lu-PSMA-617) (18). However, a loss of PSMA expression has also been noted, sometimes related with the progression of the malignancy from the androgen-dependent to the androgen-independent stage, while there are also reports for elevated PSMA expression related to androgen deprivation treatments (19, 20).

Another promising target for receptor-mediated tumor imaging and radionuclide therapy of PCa is the GRPr, also known as bombesin receptor subtype 2 (21). Upon binding of a suitable ligand, the GRPr is activated, causing several physiological functions and the control of exocrine and endocrine secretion (22). Following the successful application of radiolabeled somatostatin peptide analogs in neuroendocrine tumors for Nuclear Medicine (23), numerous radiolabeled GRPr targeting radioligands have been studied so far, both preclinically and in clinical trials, for prostate and breast cancer patients (21, 24). For the GRP receptor, two types of ligands have been identified, all derivatives of the natural bombesin peptide. The first type refers to agonistic peptide ligands of GRPr, which are known to internalize after binding, and the second to antagonistic peptide ligands, which despite showing higher cell-surface binding, do not internalize (25).

Amidst the various PCa cell lines, PSMA and GRPr expression differs. GRPr is highly expressed in androgen-independent PC3 cancer cells, while androgen-sensitive LNCaP cancer cells are GRPR negative (19). PSMA, on the other hand, is highly expressed in androgen-sensitive LNCaP cancer cells, which are negative for GRPr (26). Therefore, PSMA and GRPr are both emphasized as crucial targets to increase diagnostic precision. In order to address PCa tumor heterogeneity, given that their expression is extremely heterogeneous, the utilization of both pharmacophores has been suggested (27), specifically, the utilization of heterodimeric radiotracer ligands able to target both PSMA and GRPr (28). The first heterodimer, which was reported in 2014 (27) combined the two pharmacophores: a urea-based PSMA inhibitor, linked to a bombesin peptide agonist. However, utilizing a GRPr-targeting antagonist provides several benefits over using an agonist (29).

Nanoparticles can be designed to have multiple pharmacophores on their surface, either of the same type (homo-multivalency), or of different types (hetero-multivalency). Such functionalization can strengthen their binding and increase their affinity for the tumor, by simultaneously interacting with multiple receptors on the cell surface (30). This strategy is supported by various studies which refer to nanoparticle functionalization with multiple ligands targeting receptors such as RGD/α_ν_β_3_ integrin, VEGF/VEGF-r, bombesin, etc. (11, 31–33).

In this study, we synthesized two pharmacophores, a Lys-CO-Glu peptidomimetic targeting the PSMA receptor and a bombesin peptide targeting the GRPr. The above pharmacophores were coupled to iron oxide (Fe_3_O_4_) nanoparticles carrying thiol (IONs-SH) groups, resulting in mono-functionalized IONs-PSMA and IONs-BN and dual functionalized IONs-PSMA/BN. All nanoparticles were directly labeled with Technetium-99 m (^99m^Tc) and their purity and stability were assessed in the presence of human serum, DTPA, and histidine. Moreover, their biocompatibility in red blood cells (RBCs) was evaluated. *In vitro* studies including cytotoxicity studies, wound-healing assay, saturation and internalization experiments were carried out in prostate cancer cell lines expressing either PSMA (LNCaP cells) or GRPr (PC3 cells), to investigate their potential future use as SPECT/MRI diagnostic tools for PCa.

## Materials and methods

### General

The chemicals used for the synthesis of the nanoparticles were of analytical grade and were used without further purification unless otherwise indicated. TRITON X was purchased from Merck (Germany); Phosphate Buffer Saline Tablets (PBS) (Fischer, Belgium); Bovine Serum Albumin (BSA) fraction V, 96%—lyophilized powder from Sigma-Aldrich (Steinheim, Germany); anhydrous Tin(II) chloride (SnCl_2_) 98% from Acros Organics (Fisher Scientific, UK); Sodium Citrate from Riedel-de Haën (Seelze, Germany), and Acetone from Merck (Darmstadt, Germany). Human serum was purchased from Sigma Aldrich (St. Louis, MO, USA).

Attenuated total reflectance Fourier transform infrared (ATR-FT-IR) spectra were obtained on a Perkin Elmer Spectrum 100 Spectrometer. The spectra were scanned over the range 4,000–350 cm^−1^. Dynamic light scattering (DLS) measurements were performed for morphological characterizations, using a multipurpose titrator on a Malvern Instruments Zetasizer Nano Series. In order to achieve the best possible dispersion of the sample, an ultrasonic bath for sonication (Elma Sonic, S 30H) was used. A commercial ^99^Mo/^99m^Tc generator (Mallinckrodt Medical B.V.) was used to elute ^99m^Tc, as Na[^99m^Tc]TcO_4_^−^. A lower-activity commercial ^68^Ge/^68^Ga-generator was acquired from Eckert & Ziegler (Berlin, Germany). Radioactivity of the [^99m^Tc]Tc- and [^68^Ga]Ga-labeled complexes was measured using a dose calibrator (Capintec, Ramsey, NJ). Instant thin layer chromatography—silica gel (ITLC-SG) 60 sheets (5 cm × 10 cm) (Merck, Darmstadt, Germany) were used for the determination of radiolabeling yield/purity and *in vitro* stability studies, which were developed in the following mobile phases: sodium citrate (0.1 M), and acetone and then measured with a Radio-TLC Scanner (Scan-Ram, LabLogic, Sheffield, UK). For *in vitro* experiments and cell bound or internalized radioactivity, a gamma scintillation counter (γ-counter, Packard Cobra II, GMI, Minnesota, USA) was used. HPLC was performed using a Waters 600 Controller pump and a Waters 996 Photodiode Array detector and a γ-RAM radioactivity detector to measure radioactive flow on a Jupiter C4 Column (150 mm × 4.60 mm, 5 μm, 300 Å, Phenomenex, Torrance, CA, USA). The UV detection wavelength was set at 220 nm for all experiments.

The cancer cell lines PC3 and LNCaP were obtained from the cell bank of the Laboratory of Radiobiology, Institute of Nuclear & Radiological Sciences & Technology, Energy & Safety, NCSR “Demokritos”. The cells were free of mycoplasma contamination, as judged visually under microscope observation and by regular 40,6-diamidine-20-phenylindole dihydro-chloride (DAPI) staining of the cell cultures. The media for the cultures Dulbecco's Modified Eagle Medium (DMEM) and Roswell Park Memorial Institute (RPMI)-1640 were purchased from Biowest (Riverside, MO, USA), and the 3-(4,5-dimethylthiazol-2-yl)-2,5-diphenyltetrazolium bromide- (MTT) from Applichem (Darmstadt, Germany). Optical density measurements in the *in vitro* experiments were conducted using a LabSystems Multiskan RC Microplate Reader (Thermo Fisher Scientific, MA, USA).

### Chemical synthesis of pharmacophores PSMA and BN

**SCHEME 1:**
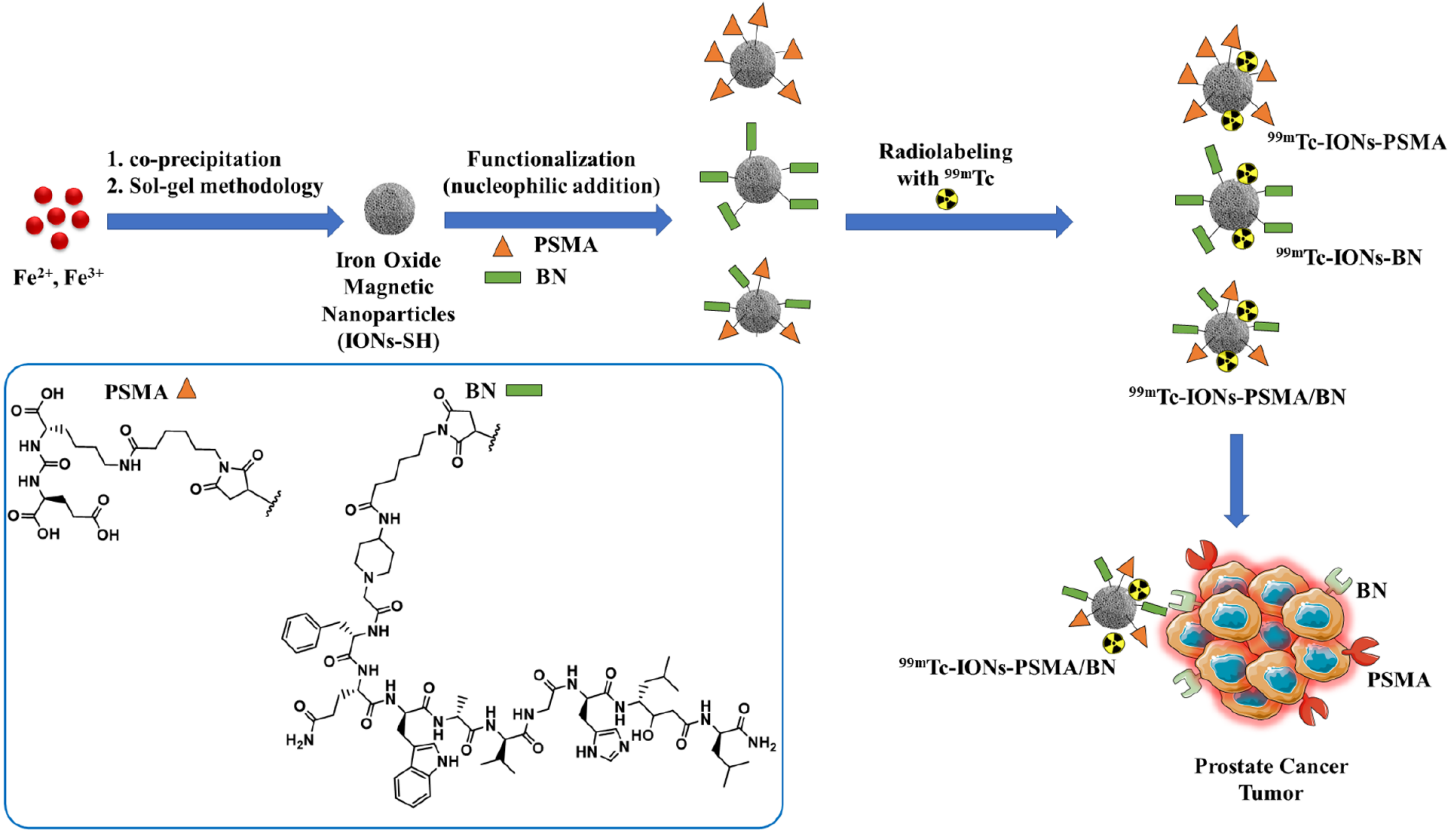
Chemical structures of the IONs functionalized with pharmacophores PSMA (IONs-PSMA), BN (IONs-BN), or both (IONs-PSMA/BN). The IONs were radiolabeled with ^99m^Tc, either with its direct complexation to the IONs surface, using anhydrous SnCl_2_ as the reducing agent of Na[^99m^Tc]TcO_4_^−^, or by using the [^99m^Tc][Tc(H_2_O)_3_(CO)_3_]^+^ precursor. The directly labeled IONs were further investigated due to their higher RCC.

The synthesis of Lys-CO-Glu (PSMA), the pharmacophore for the PSMA receptor, and the bombesin peptide (BN), 4-amino-1-carboxymethyl piperidine-[D-Phe^6^, Sta^13^]BN(6–14), the pharmacophore for the GRPr, was performed using solid-phase peptide chemistry methods according to previously published methods with appropriate modifications (34–36). Both pharmacophores were functionalized with a maleimide group to enable their attachment to the nanoparticles carrying -SH groups on their surface via nucleophilic addition, according to previously published methods (37, 38) (see also Scheme 1). The characterization of both pharmacophores has been described in detail in our previous work by Liolios et al. 2022 (37).

### Synthesis and functionalization of IONs

The IONs were synthesized using a co-precipitation method, where ferrous (Fe^2+^) and anhydrous ferric (Fe^3+^) salts were dissolved in aqueous alkaline conditions at 50°C under nitrogen atmosphere (Scheme 1) (37, 38). In order to prepare magnetite, a mixture of Fe^2+^ and Fe^3+^ chloride in a 1:2 molar ratio was dissolved in deionized water (80 ml) and stirred for 30 min under nitrogen atmosphere at 70°C. Then 30% NH_3_ aqueous solution was added to the existing mixture and the reaction was stirred for 1 h. After the completion of the reaction, the IONs were separated by an externally applied magnetic field, washed three times with H_2_O and EtOH, dispersed in deionized H_2_O, and finally stored at 4–6°C.

A sol-gel methodology, utilizing MPTES, was used to develop a thin silica layer on the surface of IONs, which then carried -SH (IONs-SH) groups on their surface and were ready for further functionalization. Specifically, a dispersion (5 ml) of IONs (*C*_Fe_ = 2.64 μmol/ml) was introduced in EtOH-H_2_O (15:25, 40 ml) and left under stirring in nitrogen-atmosphere (80°C, 30 min). Then, MPTES (125 μl) and 30% NH_3_ (375 μl) were added, resulting in a homogenous mixture, which was left overnight to react under stirring at 80°C. The final product IONs-SH was collected with the aid of an externally applied magnetic field, washed three times with EtOH and H_2_O and stored as a dispersion in H_2_O.

The IONs-SH were then conjugated with the pharmacophores PSMA and BN via nucleophilic addition according to previously published methods (37, 38). Briefly, in an aqueous suspension of IONs-SH (*C*_Fe_ = 2.71 μmol/ml) carrying the -SH groups, the pharmacophores were added (PSMA and BN: *C* = 1.0 μg/μl in PBS, pH = 7.4, reaction ratio *C*_IONs−SH_/*C*_PSMA or BN_ = 1/0.25) and the final products IONs-PSMA/BN were purified and stored in H_2_O.

The amount of bound pharmacophore was calculated by subtracting the remaining pharmacophores in the supernatant of the reaction mixture from the initial amount (control: PSMA and BN without the IONs) using an analytical RP-HPLC method to separate PSMA and BN, by integrating the area under the curve (UV-VIS detector, 220 nm—Time) of the peaks.

### Characterization of the IONs

**Figure 1:**
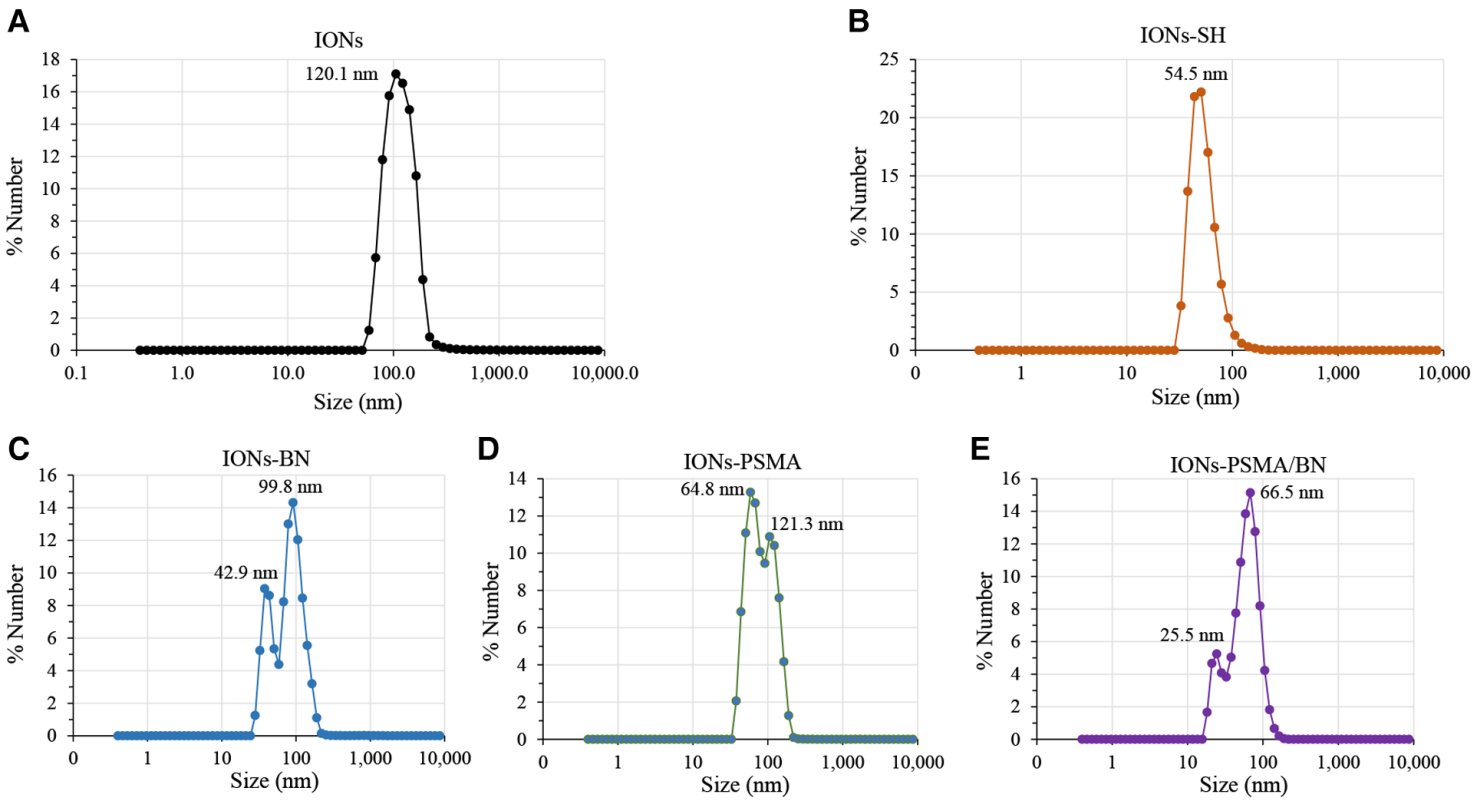
Hydrodynamic diameter of IONs measured with DLS: (**A**) before silica gel thin layer modification (IONs); (**B**) after MPTES modification (IONs-SH); and (**C–E**) after functionalization with pharmacophores BN, PSMA and PSMA/BN.

**Figure 2:**
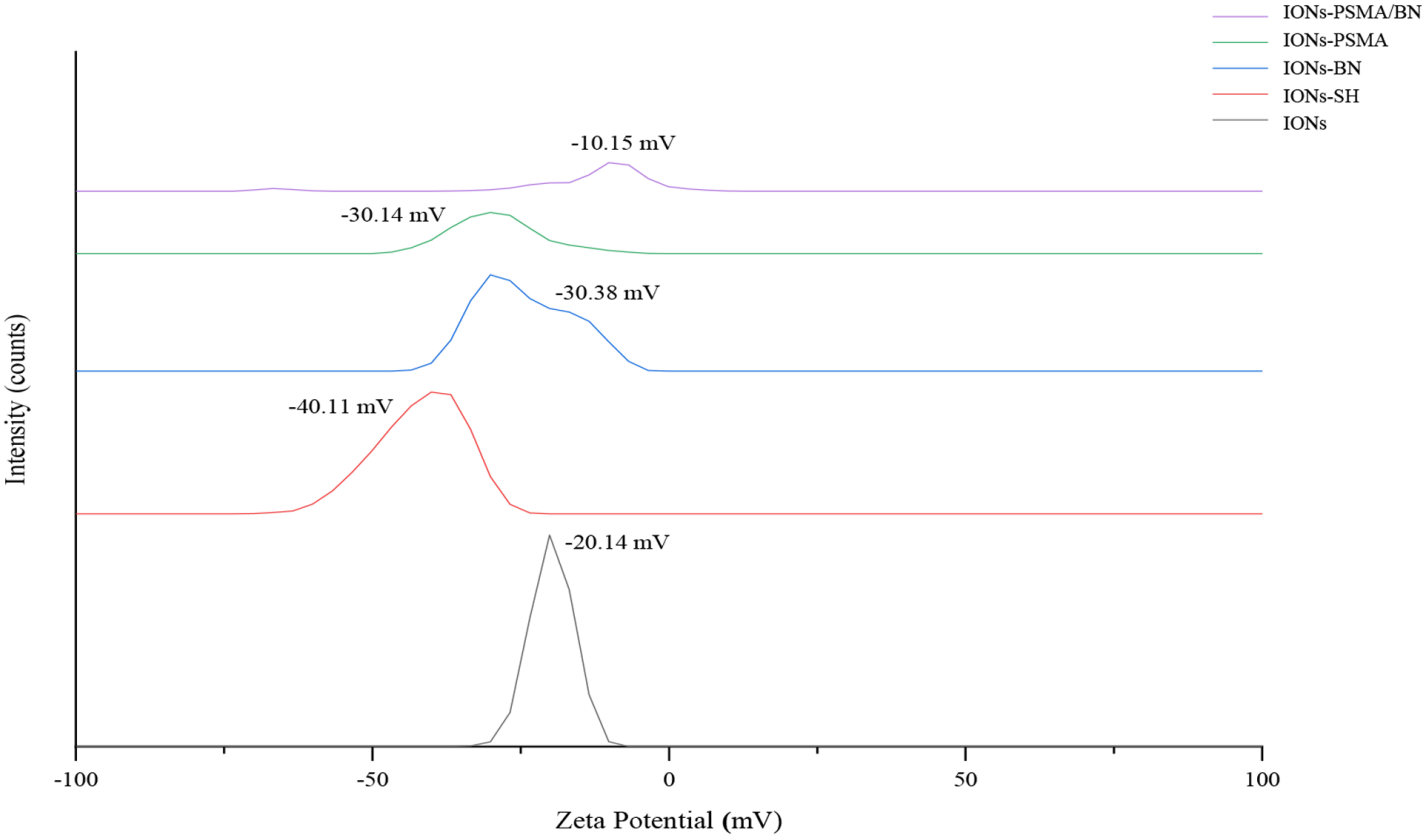
Observed changes in ζ*p* values of nanoparticles before (IONs) and after MPTES modification (IONs-SH) and after functionalization with PSMA (IONs-PSMA), BN (IONs-BN), or both PSMA and BN (IONs-PSMA/BN).

**Figure 3:**
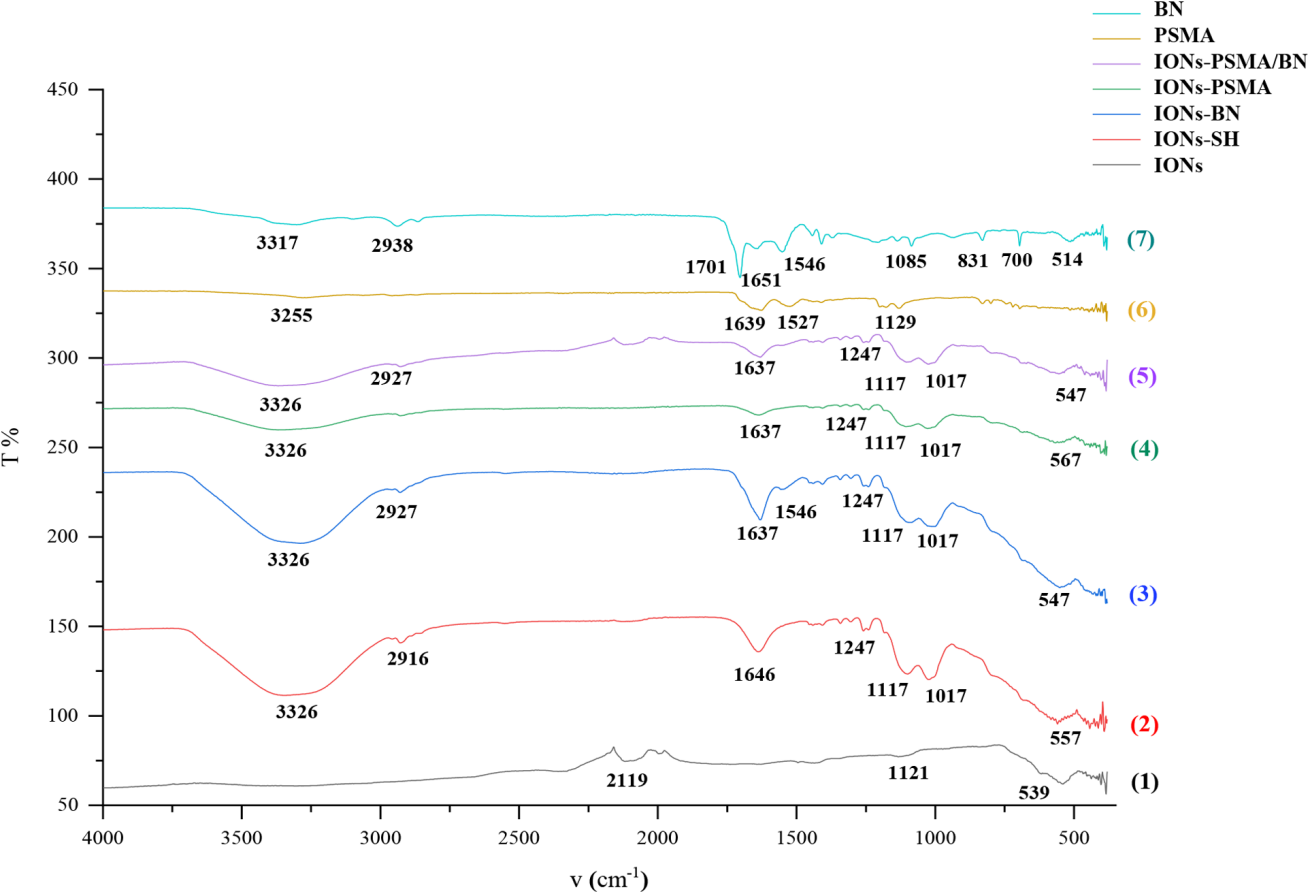
FT-IR structural characterization of compounds: (1) IONs, (2) IONs-SH, (3) IONs-BN (4) IONs-PSMA, (5) IONs-PSMA/BN, (6) PSMA, and (7) BN.

The nanoparticles IONs, IONs-SH, IONs-PSMA, IONs-BN, and IONs-PSMA/BN were characterized for their size and zeta potential (ζ*p*), using DLS and FT-IR according to standard procedures (32, 39, 40). Briefly, the DLS experiments (Figure 1) showed that their size ranged between 25.5 and 121.3 nm, and it remained small even after their functionalization (e.g., IONs-SH: 54.5 nm, IONs-PSMA: 64.8 nm), possibly due to the small size of the bound peptides. The zeta potential (ζ*p*) measurements (Figure 2) showed negative ζ*p*, which was altered after each functionalization step: (a) IONs, ζ*p* = −20.14 mV, (b) IONs-SH, ζ*p* = −40.11 mV (c) IONs-PSMA, ζ*p* = −30.38 mV, (d) IONs-BN, ζ*p* = −30.14 mV, (e) IONs-PSMA/BN, ζ*p* = −10.15 mV. The FT-IR analysis (Figure 3) compared the non-functionalized IONs, the pharmacophores PSMA & BN and the functionalized IONs and the appearance of characteristic peaks, i.e., S–H bonds, 1,646 cm^−1^, or C–H, 2,957 cm^−1^, provided proof for their functionalization.

### Radiolabeling and radiochemical analysis of IONs

#### ^99m^Tc-labeling

Two methods of radiolabeling with ^99m^Tc were tested for the various types of IONs, either with a reduction of Tc(VII) to Tc(V) and then direct complexation to the IONs surface or with the use of the tricarbonyl precursor [^99m^Tc][Tc(H_2_O)_3_(CO)_3_]^+^.

The first approach of direct ^99m^Tc-radiolabeling was performed using anhydrous SnCl_2_ as the reducing agent. Initially SnCl_2_ (8.0–9.0 mg) was dissolved in HCl (>37%) (250 μl), and subsequently, deionized water was added to achieve a total volume of 5 ml (Solution A). Then, 500 μl of phosphate buffer saline (PBS) (pH = 10) was mixed with each nanoparticle (IONs-SH, IONs-PSMA, IONs-BN, and IONs-PSMA/BN: 50 μl, 7.56 μg, *C* = 2.71 μmol/ml). Afterwards, Solution A (50 μl) was added to the mixture, and the pH was adjusted to pH = 6.5–7.0. Finally, Na[^99m^Tc]TcO_4_^−^ (100 μl, 0.3–2.53 mCi) was added and incubated for 1 h at 75°C (final reaction volume = 0.7 ml, *C*_IONs_ = 10.81 μg/ml). The directly [^99m^Tc]Tc-labeled IONs were then used without further purification, provided their RCC was above 94%.

The percentage of radiochemical conversion (% RCC) was determined by two ITLC-SG systems using acetone and a sodium citrate solution (0.1 M) as the mobile phases. The [^99m^Tc]Tc-IONs reaction mixture (5 μl) was spotted on an ITLC-SG strip (1.5 cm × 10 cm), allowed to dry and then developed until the solvent reached the front. The acetone moves the free [^99m^Tc]^99m^TcO_4_^−^ to the front (*R_f_* = 0.8–1.0) of the ITLC strip leaving the reduced/hydrolyzed ^99m^Tc along with the labeled complex (^99m^Tc-NPs) at the origin (*R_f_* = 0.0–0.2). Colloid formation was also determined by ITLC-SG using sodium citrate 0.1 M, where the radiocolloids remained at the origin (*R_f_* = 0.0–0.2), while both free ^99m^TcO_4_^−^ and ^99m^Tc-labeled complex migrated to the front (*R_f_* = 0.8–1.0). The TLC strips of both systems were cut in half and measured with a gamma counter. With the combination of these two systems, we calculated the percentage of the produced ^99m^Tc-NPs under the above conditions, using the following equation (Eq. 1):

(1) $$\text{\%}\mathrm{RCC}={\text{\%}}^{99m}\mathrm{Tc}\text{-}\mathrm{labeled}\;\mathrm{complex}\;\text{-}\;\text{\%}\mathrm{colloida}l^{99m}\mathrm{Tc}$$

The [^99m^Tc][Tc(H_2_O)_3_(CO)_3_]^+^ precursor is prepared by reducing [^99m^Tc]TcO_4_^−^ ions, which are obtained from the ^99^Mo/^99m^Tc generator, in the form of Na[^99m^Tc]TcO_4_^−^ solution, in an atmosphere saturated with CO as described in literature (41, 42). The formation of [^99m^Tc][Tc(H_2_O)_3_(CO)_3_]^+^ is analyzed by Reverse Phase—High-Performance Liquid Chromatography (RP-HPLC). RP-HPLC is performed with an elution solvent system consisting of MeOH/0.1% trifluoroacetic acid (TFA) and H_2_O/0.1% TFA (*R_t_* [^99m^Tc][Tc(H_2_O)_3_(CO)_3_]^+^: 4–6 min; *R_t_* [^99m^Tc]TcO_4_^−^: 3.0 min). The radiochemical conversion (RCC) was measured >95%.

Iron oxide nanoparticles (50 μl) were radiolabeled with [^99m^Tc][Tc(H_2_O)_3_(CO)_3_]^+^ (100 μl, 0.61–1.44 mCi) and incubated at 75°C for 1 h. The percentage of radiolabeling yield was determined by ITLC-SG using acetone as the mobile phase. With the same procedure as described above, the reaction mixture (5 μl) was spotted on an ITLC-SG strip (1.5 cm × 10 cm), allowed to dry and then developed in acetone. The free [^99m^Tc]TcO_4_^−^ migrates to the front (*R_f_* = 0.8–1.0) of the ITLC strip leaving the labeled complex ([^99m^Tc]TcO_4_^−^ -NPs) at the origin (*R_f_* = 0.0–0.2). The radio-activity on the ITLC-SG strips was visualized using a Radio-TLC Scanner. Data collection and analysis were performed with Laura software v. 5.0.4.29.

#### *In vitro* stability study

The *in vitro* stability of the [^99m^Tc]Tc-labeled complexes of IONs (IONs-SH, IONs-PSMA, IONs-BN, IONs-PSMA/BN) was evaluated in human serum up to 2 h post-radiolabeling (p.r.), and in histidine and DTPA up to 24 h p.r. The IONs used for the stability studies were radiolabeled directly using anhydrous SnCl_2_ as the reducing agent of Na[^99m^Tc]TcO_4_^−^. Each labeled complex was incubated with serum at a ratio of 1:9 (v/v) at 37°C. A sample (5 μl) was then extracted and spotted on an ITLC-SG strip and analyzed by using acetone as the mobile phase. With this system, the [^99m^Tc]Tc-IONs remain at the origin (*R_f_* = 0.0–0.3), while [^99m^Tc]TcO_4_^−^ migrates to the front (*R_f_* = 0.7–1.0).

For the other stability studies, fresh solutions of histidine and DTPA were prepared in water, at a concentration of 0.01 M. An aliquot of [^99m^Tc]Tc-IONs (500 μl) was added to histidine and DTPA solutions (500 μl), respectively and incubated at 37°C. Samples (5 μl) were extracted from the mixture at *t* = 0, 1, 3, and 24 h, and analyzed by ascending ITLC-SG using acetone and saline as mobile phases. With acetone as the mobile phase the labeled IONs remain at the origin (*R_f_* = 0.0–0.3), while [^99m^Tc]TcO_4_^−^ migrates to the front (*R_f_* = 0.7–1.0); with saline the labeled IONs and [^99m^Tc]TcO_4_^−^ remain at the origin (*R_f_* = 0.0–0.3), while the [^99m^Tc]Tc-histidine and [^99m^Tc]Tc-DTPA complexes migrate to the front (*R_f_* = 0.7–1.0) (39).

#### Hemolysis assay in red blood cells

The biocompatability of the IONs (IONs-SH, IONs-PSMA, IONs-BN and IONs-PSMA/BN) was evaluated using a hemolysis assay, with red blood cells (RBCs) isolated from human blood samples, according to previously published methods (43–45). Blood samples were collected from healthy donors in heparinized polypropylene tubes. The donors participating in this study provided their consent for the use of their blood samples in these experiments and underwent blood sampling. All experiments were carried out in accordance with relevant guidelines and regulations and were performed in triplicate.

In brief, blood was centrifuged at 3,000 rpm for 15 min to separate the plasma from RBCs. The RBCs were then washed three times with PBS (0.01 M, pH 7.4) free of magnesium and calcium and the last supernatant was stored. A sample (285 μl) of the IONs (*C* = 0.1, 1, and 10 μg/ml), was added to RBCs (15 μl). An equal volume of PBS from the last supernatant (285 μl) was mixed with RBCs (15 μl) and used as a Negative control sample, while TRITON X (10 μl, 10%) along with PBS (275 μl) and RBCs (15 μl) were used as a Positive control sample. The samples together with the control samples were incubated for 3 h at 37°C and then centrifuged once more at 3,000 rpm for 5 min. Finally, a sample of the supernatant (100 μl) was taken from each sample and placed in 96-well plates. The absorbance was measured at 450 nm in a LabSystems Multiskan RC Microplate Reader, while the hemolysis ratio for each case was calculated according to the following equation (Eq. 2) (44):

(2) $$\text{\%}\;\mathrm{Hemolysis}=\frac{\left(OD_{\mathrm{IONPs}}\;\text{–}\;OD_{\mathrm{Negative}\;\mathrm{control}}\right)}{\left(OD_{\mathrm{Positive}\;\mathrm{control}}\;\text{–}\;OD_{\mathrm{Negative}\;\mathrm{control}}\right)}$$

### *In vitro* evaluation in LNCaP and PC3 cells

#### Cell culture

Cell studies were performed with the BN-positive PC3 cells (bone metastasis, grade IV prostatic adenocarcinoma, ATCC CRL-1435) and PSMA-positive LNCaP cells (ATCC CRL-1740). PC3 cells were cultured in DMEM medium, while LNCaP cells were cultured in RPMI medium. Both culture media were supplemented with 10% fetal bovine serum (FBS, Вiowest, S1400, EU) and 2 mM L-glutamine (Вiowest, EU). Cells were incubated in a controlled humidified atmosphere containing 5% CO_2_ at 37°C and sub-cultured weekly after being harvested from the flask surface by trypsin/ethylenediaminetetraacetic acid (EDTA) solution (0.25% trypsin/0.02% EDTA in PBS, Biowest, EU).

#### Cytotoxicity study

For the determination of the cytotoxicity of the nanoparticles (IONs-SH, IONs-PSMA, IONs-BN, and IONs-PSMA/BN), the 3-(4,5-dimethylthiazol-2-yl)-2,5-diphenyltetrazolium bromide (MTT method) was used, which is based on the reduction of MTT, a yellow water-soluble salt of tetrazole, to purple formazan crystals in living cells. The metabolic activity of the cells is proportional to the color density of the purple formazan crystals and indicates cell viability and toxicity of the substances incubated with the cell (42, 46).

The PC3 and LNCaP cancer cell lines were used for this assay. Approximately 15 × 10^3^ cells per well in 100 μl of the medium are cultured in 96-well plates and incubated for 24 h at 37°C in a 5% CO_2_ incubator. After incubation, the nanoparticles were added to the wells in five concentrations (*C* = 0.01, 0.1, 1, 10, 20 μg/ml). In the control group, only the medium was added (DMEM for PC3 cells and RPMI for LNCaP cells). The cells were then incubated with the derivatives for 24 h. After 24 h, the medium was removed and MTT (100 μl, *C*_MTT_ = 1 mg/ml) was added to each well and then incubated for another 4 h. The formazan crystals formed were solubilized in isopropanol (100 μl). The absorbance is measured at 540 nm in the ELISA reader. The results were expressed as the percentage of viable cells (Viability %) (Eq. 3) (42). All experiments were performed in quadruplicates and were repeated 4 times.

(3) $$\mathrm{Viability}\;\text{\%}=100\frac{OD_{\mathrm{sample}}}{OD_{\mathrm{control}}}$$

where OD_sample_: optical density of the cells incubated with the derivatives under study, and OD_control_: optical density of the cells with incubated medium only.

#### Scratch/wound healing assay

The wound healing assay was performed to study the effects of the synthesized nanoparticles (IONs-SH, IONs-PSMA, IONs-BN and IONs-PSMA/BN) on the cellular migration and proliferation of PC3 and LNCaP cell lines (47, 48). Approximately 2 × 10^4^ PC3 cells were seeded in two 12-plates and incubated for 24 h until they reached confluency. A scratch was grafted in each well using a pipette tip and each well was washed with PBS to remove cellular debris. The same method was used for LNCaP cells, but due to their slower proliferation rate the experiment was performed in a 24-well plate. After creating the scratch, the different types of nanoparticles were added in triplicate wells. The same concentration (*C* = 10μg Fe/ml in DMEM for PC3 and in RPMI for LNCaP) was selected to monitor the healing of the wound at different time points (0, 24, 48 and 72 h) with an Inverted Microscope (EXI-310 series, Accu-Scope Inc. & Unitron). Approximately 10–15 pictures were captured from each well following the entirety of each scratch, to include possible statistical deviations.

The quantification of the wound closure (WC) is performed by image analysis through the software ImageJ and is expressed according to the equation (Eq. 4):

(4) $$\text{\%}\;WC_{t_{i}}\;=100\frac{\left(A_{t_{0}}-A_{t_{i}}\right)}{A_{t_{0}}}\;$$

where (*t*_0_) was right after the addition of the samples and (*t_i_*) the different time points. A two-way ANOVA statistical analysis with multiple comparisons was performed using the Tuckey test to evaluate the significance of the results within each sample for the different time points [*p* .12 (ns), *p* .033 (*), .002(**), <.001(***)].

#### Cell binding studies

Cell binding studies were conducted in 24 well plates of PC3 (∼5 × 10^4^/well) and LNCaP cells (∼2 × 10^5^/well) 1 day after seeding with six different concentrations of directly radiolabeled [^99m^Tc]Tc-IONs (IONs-SH, IONs-PSMA, IONs-BN, IONs-PSMA/BN, *C* = 0.5, 1, 2, 3, 4, 5 μg/ml in *V*_incubation_ = 1.0 ml), at 4°C and 37°C for 45 min, according to protocols previously published by Liolios et al. 2022 (37). Both cell lines were incubated with [^99m^Tc]Tc-IONs-SH (negative control) and [^99m^Tc]Tc-IONs-PSMA/BN. The PSMA positive LNCaP cells were also treated with [^99m^Tc]Tc-IONs-PSMA, while the GRPr positive PC3 cells with [^99m^Tc]Tc-IONs-BN (positive controls).

The medium was removed and the cells were first washed with PBS and then with an acidic buffer (Glycine/HCl), in order to determine the surface binding of the nanoparticles on the cancer cells. Internalized radioactivity was determined by lysing the cells with NaOH (1.0 M). The acid wash and lysates were collected, and the radioactivity was measured using a γ-counter (37).

### Statistical analysis

Statistical analysis was conducted with Graph Pad Prism using the ordinary Two-way ANOVA tests (Dunnett's multiple comparisons test), alpha = 0.05, where (ns) *p* > 0.05, (*) *p* ≤ 0.05, (**) *p* ≤ 0.01, (***) *p* ≤ 0.001, (****) *p* ≤ 0.0001.

## Results and discussion

### Chemical synthesis

The IONs were synthesized utilizing co-precipitation of ferrous (Fe^2+^) and anhydrous ferric (Fe^3+^) salts in aqueous alkaline conditions (IONs) (38). Then sol-gel methodology was used to develop a thin silica layer on their surface with MPTES, carrying -SH groups, and resulting in IONs-SH, respectively. Iron concentration was calculated through the phenanthroline protocol (49), and was found to be *C*_Fe_ = 2.64 μmol/ml.

Further on, the IONs were conjugated with pharmacophores (PSMA) and (BN) via nucleophilic addition (Michael reaction), affording mono-functionalized IONs-PSMA, IONs-BN and dual-functionalized IONs-PSMA/BN (Scheme 1). Chemical synthesis of the Lys-CO-Glu derivative (PSMA, total synthesis yield 35%) and the bombesin analogue (BN, total synthesis yield 52%) (Scheme 1) was accomplished using the Fmoc strategy of solid-phase peptide synthesis (SPPS) (35, 37) and were chemically characterized with RP-HPLC and Maldi-MS and ^1^H and ^13^C NMR (37). Both pharmacophores were coupled to maleimidohexanoic acid (at their N-terminal) to facilitate the functionalization of nanoparticles carrying the -SH groups on their surface under mild conditions. The iron concentration of the final products (IONs-PSMA, IONs-BN, and IONs-PSMA/BN) was calculated *C*_Fe_ = 2.71 μmol/ml.

### Characterization of IONs

According to previous studies, this type of IONs has a spherical shape and a small size (size range: 10 ± 2 nm) (38). The results of DLS experiments determining the hydrodynamic diameter of the IONs before and after their functionalization (IONs, IONs-SH, IONs-PSMA, IONs-BN, IONs-PSMA/BN) are summarized in Figure 1. The size of the nanoparticles ranged between 25.5 and 121.3 nm, and it remained small even after their functionalization (e.g., IONs-SH: 54.5 nm, IONs-PSMA: 64.8 nm), possibly due to the small size of the bound peptides. The larger size of non-functionalized IONs (120.1 nm) can be explained by the absence of hydrophilic functional groups on their surface, which at pH 7.0 leads to the formation of aggregates, while on the other hand, the introduction of peptides on their surface forms a protein corona around them (50). After each synthetic step the zeta potential (ζ*p*) was measured for the IONs and the results are summarized in Figure 2. The alterations in the ζ*p* pattern indicate a successful functionalization: (a) the initial negative ζ*p* (ION, ζ*p* = −20.14 mV) became more negative (IONs-SH, ζ*p* = −40.11 mV) after the incorporation of the silica -SH groups (MPTES) and less negative after functionalization with PSMA (ζ*p* = −30.38 mV) or with BN (ζ*p* = −30.14 mV) or with both (ζ*p* = −10.15 mV); The ζp of the final products was clearly affected by the charges of the pharmacophores coupled to the -SH groups on their surface.

The amount of bound pharmacophore was calculated using an analytical RP-HPLC analysis of the supernatant. RP-HPLC analysis showed that pharmacophores PSMA and BN were successfully bound on the surface of IONs-SH. More specifically, for the monomeric IONs: IONs-PSMA, 41% of the added quantity (Qt) was bound and for IONs-BN, 51% of the added Qt was bound, while for the dimeric IONs-PSMA/BN, bound percentages were 40% and 60% respectively. The concentration of IONs for the reaction was *C*_IONs_ = 2.71 μmol/ml. In a reaction mixture of 1.0 ml the amount of the PSMA pharmacophore was *L*_PSMA_ = 2.71/0.25 = 10.84 μmol. If we hypothesize that the distribution of PSMA ligands was homogenous for the population of IONs after the coupling reaction, then each μmol of IONs had (0.41 × 10.84)/2.71 = 1.64 μmol of PSMA (IONs-PSMA), (0.51 × 10.84)/2.71 = 2.04 μmol of BN (IONs-BN), and (0.40 × 10.84)/2.71 = 1.6 μmol of PSMA and (0.60 × 10.84)/2.71 = 2.4 μmol of BN (IONs-PSMA/BN).

An FT-IR analysis was carried out for PSMA, BN and IONs-SH, IONs-PSMA, IONs-BN, IONs PSMA/BN, and the results of the analysis are presented in Figure 3. The peaks around 2,957 cm^−1^ were assigned to the C–H stretching and were common in all spectrums: IONs-SH and IONs-PSMA/BN or PSMA/BN. The characteristic peaks around 557 cm^−1^ (IONs-SH) are attributed to the Fe–O–Fe bond vibration of magnetite. The strong peaks and 1,247 and 1,017 cm^−1^ in IONs-SH were due to Si–O–Si and Si–O stretching vibrations, confirming the interactions between hydroxyl groups on the IONs surface and the alkoxysilane molecules: MPTES respectively. The broad band at 3,326 cm^−1^ was attributed to O–H stretching and to v–OH and –OH vibrations of intramolecular H_2_O, while the peak at 2,916 cm^−1^ to the absorption of the C–H stretching vibration. The peak at 1,646 cm^−1^ in IONs-S was related to the S–H bond. In conclusion, the FT-IR measurements confirmed the functionalization with organic moieties of the IONs (32, 38, 51).

### ^99m^Tc-labeling and radiochemical analysis of IONs

**Figure 4:**
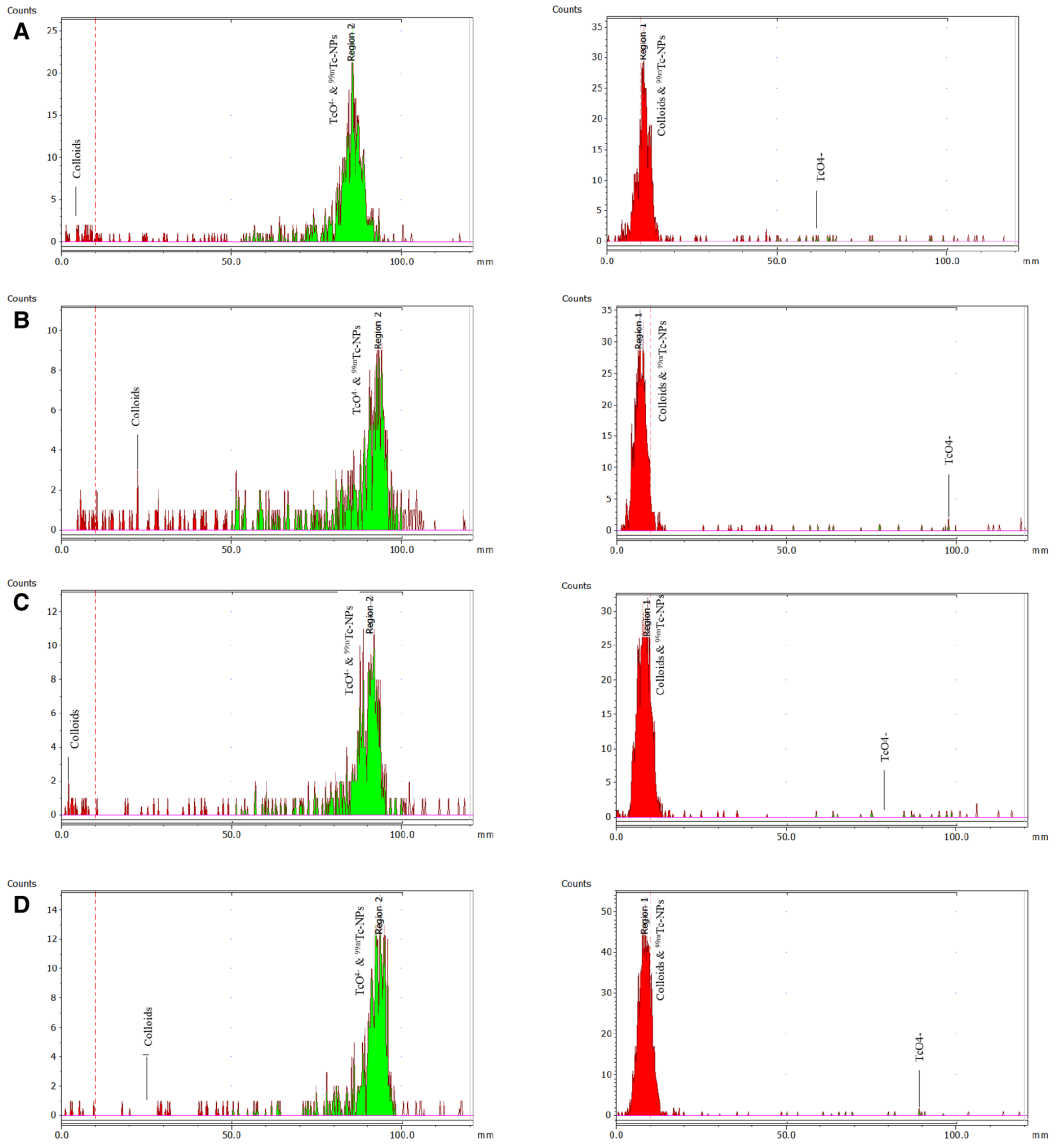
ITLC-SG radiochemical analysis (mobile phase: sodium citrate 0.1 M) to determine colloids on the left and with acetone to determine free pertechnetate [^99m^Tc]TcO_4_^−^ on the right. The analysis refers to the direct complexation of ^99m^Tc to the IONs surface for: (**A**) [^99m^Tc]Tc-IONs-SH, (**B**) [^99m^Tc]Tc-IONs-PSMA, (**C**) [^99m^Tc]Tc-IONs-BN, and (**D**) [^99m^Tc]Tc-IONs-PSMA/BN.

The RCC of the ^99m^Tc-radiolabeled samples labeled by the direct method was found to be greater than 97%, whereas the RCC of the [^99m^Tc][Tc(H_2_O)_3_(CO)_3_]^+^ radiolabeled samples was found to be greater than 75%, without any further purification (Figure 4). It was confirmed that the direct radiolabeling method is the best approach regarding ^99m^Tc-radiolabeling, and this was used for the further tests. The results of both labeling methods expressed as mean ± SD are presented in Table 1.

**Table 1:** Average RCC % ± SD for each nanoparticle for the two ^99m^Tc-labeling methods used.

	RCC %			
IONs Labeling Method	IONs -SH	IONs-PSMA	IONs-BN	IONs-PSMA/BN
Na[^99m^Tc]TcO_4_- + SnCl_2_	94.09 ± 1.43	95.05 ± 2.26	95.95 ± 1.63	96.18 ± 1.98
[^99m^Tc][Tc(H_2_O)_3_(CO)_3_]^+^	95.24 ± 1.12	73.28 ± 1.18	89.64 ± 2.74	75.86 ± 1.33

### Human serum, histidine, DTPA stability studies

Serum stability determination of the radiolabeled complexes is an important parameter because the proteins that are contained in serum may chelate ^99m^Tc, thus affecting the stability of the complex. The stability study conditions were chosen to closely resemble the body's *in vivo* environment (presence of serum, physiological temperature and pH). The results expressed as means ± SD are summarized in Table 2. The high *in vitro* stability of the directly radiolabeled complexes in serum after incubation for 1 h (over 96% for all IONs tested) reflects their stability in the biological environment upon *in vivo* administration. Further incubation (2 h) shows that ^99m^Tc is only slightly detached from the complexes, in favor of the groups present on serum proteins. Human serum stability was in the same range as previously reported (32).

**Table 2:** Average % ± SD of intact ^99m^Tc radiolabeled nanoparticles (direct method) after incubation with human serum.

	Time point (h)		
IONs	0	1	2
IONs-SH	99.72 ± 0.087	99.44 ± 0.093	99.18 ± 0.17
IONs-PSMA	97.54 ± 0.42	99.13 ± 0.22	98.99 ± 0.30
IONs-BN	98.05 ± 0.48	96.28 ± 0.50	96.36 ± 0.55
IONs-PSMA/BN	98.48 ± 12.65	98.29 ± 0.25	98.05 ± 0.33

The directly labeled nanoparticles were stable up to 24 h in the presence of both histidine (∼80% intact) and DTPA (∼95% intact), results which are comparable to those reported for ^99m^Tc-NBRh1 (39). The low transchelation degree of the complex after incubation of histidine and DTPA indicates that neither competitor could chelate ^99m^Tc, implying a high degree of stability (Table 3).

**Table 3:** Average % ± SD of intact ^99m^Tc radiolabeled nanoparticles (direct method) after incubation with histidine and DTPA.

IONs	Time point (h)	Histidine	DTPA
IONs-SH	0	99.17 ± 0.42	98.93 ± 0.21
	1	99.57 ± 0.32	99.33 ± 0.22
	3	99.78 ± 0.18	98.82 ± 0.17
	24	76.99 ± 2.01	97.19 ± 0.18
IONs-PSMA	0	99.36 ± 0.47	99.50 ± 0.20
	1	99.61 ± 0.20	99.74 ± 0.08
	3	99.70 ± 0.19	98.02 ± 0.18
	24	72.01 ± 2.00	96.62 ± 0.14
IONs-BN	0	99.32 ± 0.38	99.29 ± 0.33
	1	98.75 ± 0.22	99.63 ± 0.27
	3	99.76 ± 0.13	98.19 ± 0.11
	24	71.58 ± 2.03	96.72 ± 0.21
IONs-PSMA/BN	0	99.34 ± 0.33	99.54 ± 0.19
	1	99.80 ± 0.08	99.69 ± 0.15
	3	99.78 ± 0.21	99.32 ± 0.14
	24	75.60 ± 0.70	95.38 ± 0.26

### Hemolysis assay in red blood cells

**Figure 5:**
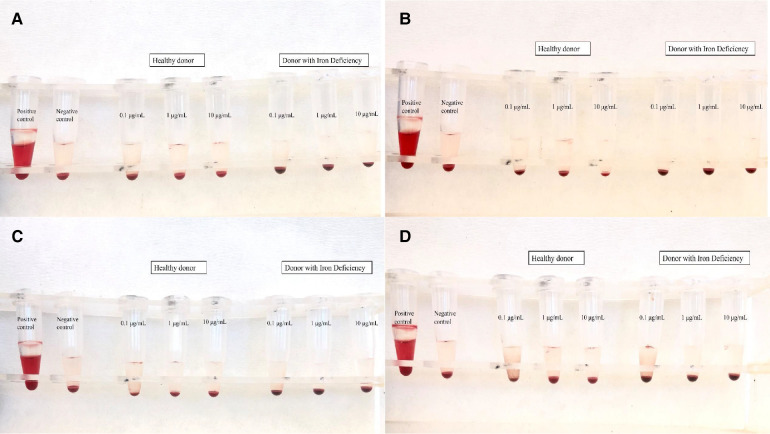
Results of hemolysis assay for (**A**) IONs-SH, (**B**) IONs-PSMA, (**C**) IONs-BN, and (**D**) IONs-PSMA/BN.

Toxic products change the tonicity of RBCs, which leads to either the hypertonic or hypotonic phenomenon. In the present study, the range of the hemolysis ratio was 0.0%–0.1%, which means that there was no indication of hemolysis (Figure 5). These results correspond with previously published works of our team on IONs functionalized with APTES (3-triethoxysilylpropylamine) and then with PSMA and BN (37), which also showed low hemolysis levels (∼5%), even after 3 h of treatment (NPs concentration *C* = 10 μg/ml).

### *In vitro* evaluation in LNCaP and PC3 cells

#### Cytotoxicity study

**Figure 6:**
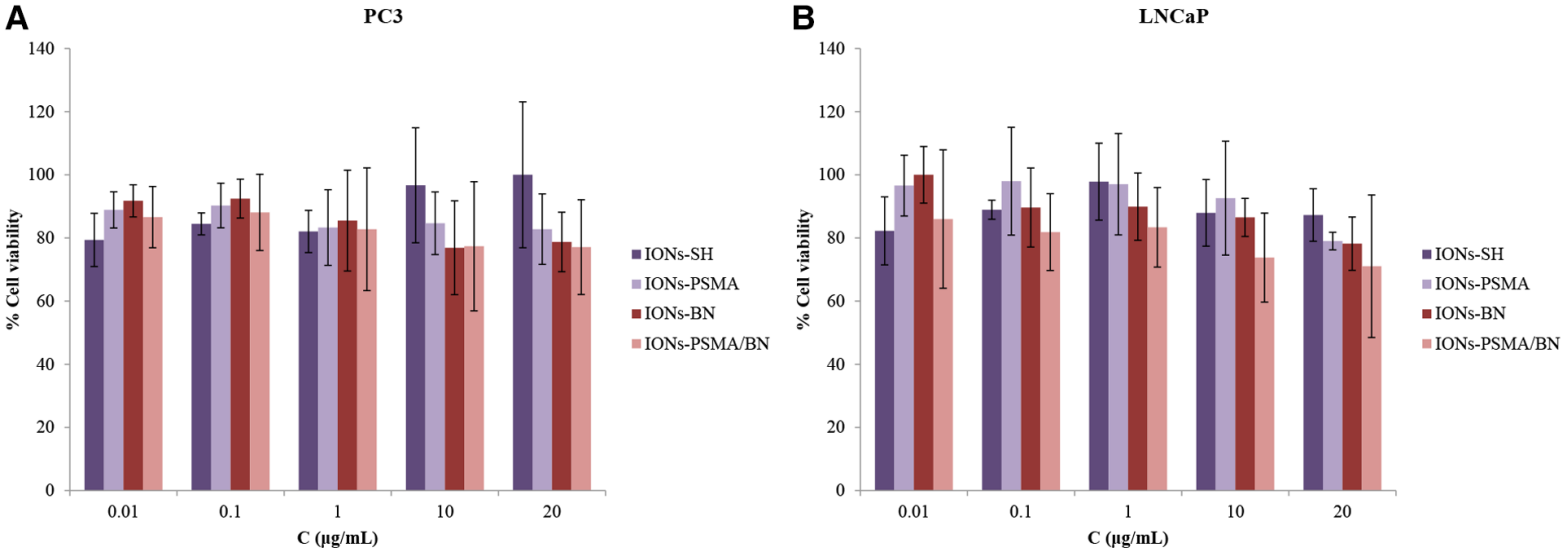
(**A**) Evaluation of cell viability via MTT assay in PC3 and (**B**) in LNCaP cells for IONs-SH, IONs-PSMA, IONs-BN, and IONs-PSMA/BN.

Regarding PC3 cells, the cell viability was above 76%, and over 71% for LNCaP cancer cells, even at the highest IONs concentration (20 μg/ml). Specifically, cell viability before functionalization (IONs-SH) with the pharmacophores was the same for both cell lines and ranged between 80% and 100%, for all concentrations tested (Figure 6). Small differences were observed after nanoparticle functionalization with either one or both pharmacophores (PSMA and BN) in comparison to the controls (IONs-SH). A slight decrease in viability (∼80%) was observed for PC3 cells, for the higher concentrations tested, possibly due to the anti-proliferating properties of the BN antagonist (52), which binds to overexpressed GRP receptors on the surface of the cells. Similar results have been reported from our team in previous studies for IONs initially functionalized with APTES (3-triethoxysilylpropylamine) and then with PSMA and BN of (37).

#### Scratch/wound healing assay

After 24 h incubation with IONs-PSMA, IONs-BN, and IONs-PSMA/BN, PC3 cells present an extremely significant WC increase of ∼30%–40%, comparable to the control, while IONs-SH present a significant increase of 21%. After 48 h all samples present an extremely significant wound healing rate, with slight differences among the samples, where IONs-BN and IONs-PSMA/BN manifest a slightly delayed healing compared to the other samples. The experiment concludes at 72 h, where most of the interactions are considered non-significant as all samples, including control, have reached >90% WC, except IONs-PSMA/BN which remained at 85%. According to the statistical analysis, the type of sample does not pose a significant factor to the % WC at each time point, but it is considered significant for the overall result.

**Figure 7:**
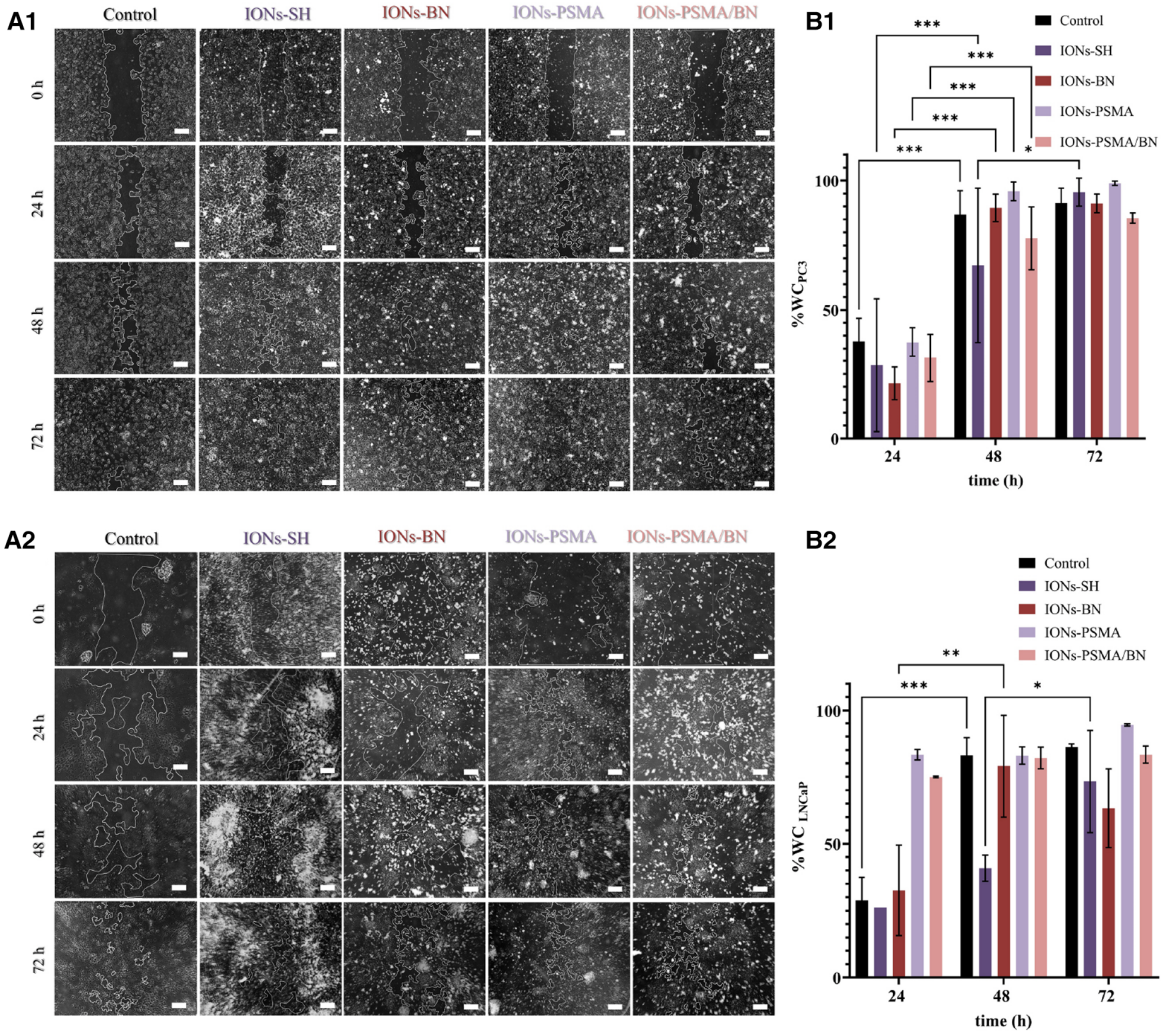
(**A1,A2**) Representative optical microscopy pictures of the scratch on PC3 and LNCaP cells (scale bar at 200 μm) and (**B1,B2**) quantified results of the wound closure (% WC). The scratch was monitored at different time points *t* = 0, 24, 48 and 72 h after incubation with IONs-SH, IONs-PSMA, IONs-BN, and IONs-PSMA/BN at *C* = 10 μg_Fe_/ml.

Concerning the effect of the samples after incubation with LNCaP cells, there was a significant increase in % WC after 24 h for IONs-SH and IONs-BN comparable to the control, while IONs-PSMA and IONs-PSMA/BN presented an extremely significant decrease of the scratch. After 48 h all samples reached ∼80% WC except IONs-SH, which demonstrated a more sustained proliferation and migration profile. Notably, after 72 h, IONs-BN presented a decrease in % WC of around 15%, in contrast to the rest of the samples (Figure 7). The delayed wound healing observed for the IONs carrying the BN antagonist were expected due to its known anti-proliferating activity especially for PC3 cells, which express the GRPr receptors.

#### Cell binding studies

**Figure 8:**
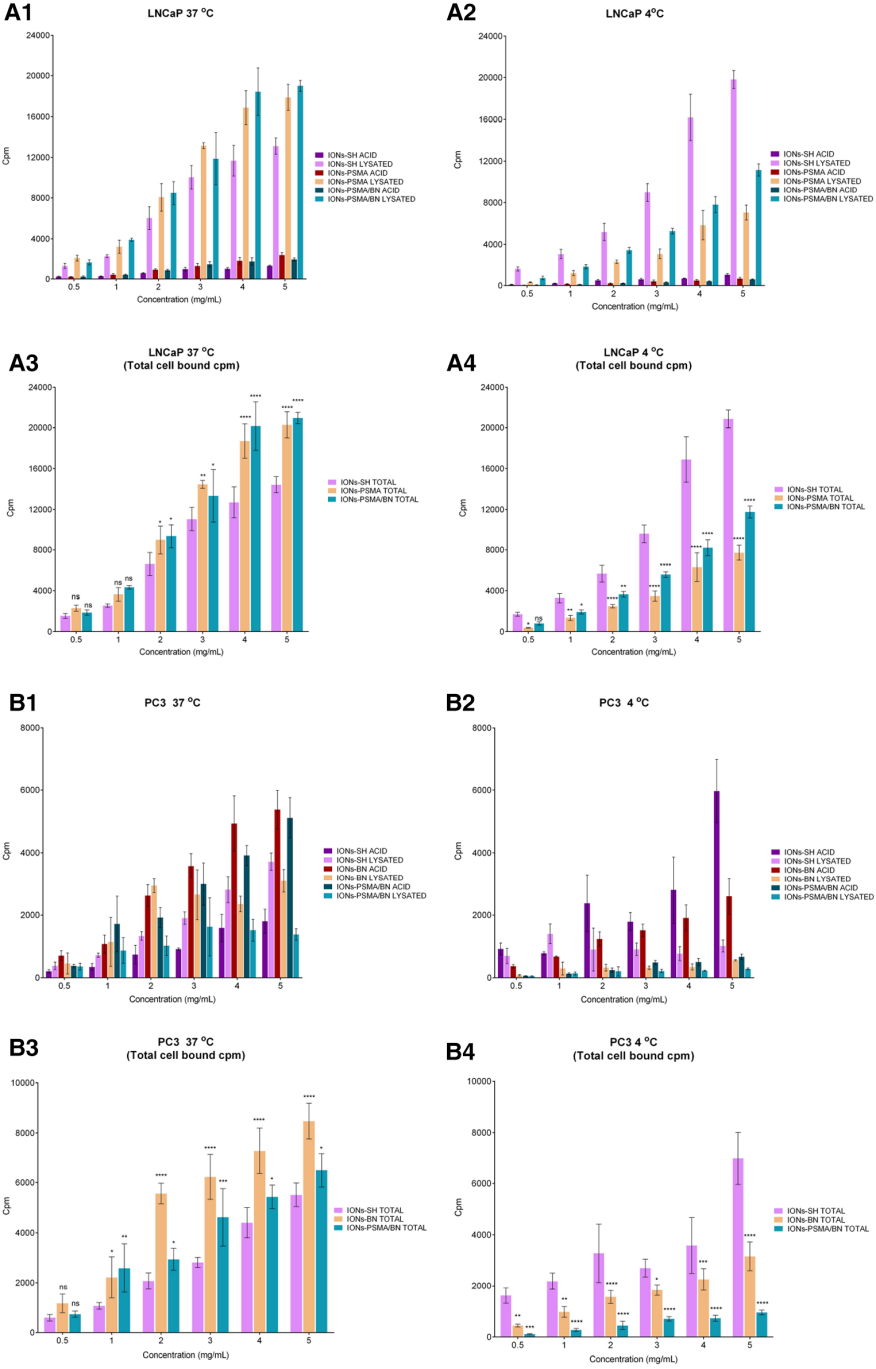
Cell binding studies on LNCaP (**A1–A4**) and PC3 (**B1–B4**) cancer cells at 37°C and 4°C for the direct complexation of ^99m^Tc to the IONs surface, [^99m^Tc]Tc-labeled: IONs-SH (control), IONs-PSMA (LNCaP), IONs-BN (PC3) and IONs-PSMA/BN (LNCaP/PC3), where ACID represents the measured radioactivity in the acid wash (surface bound) and LYSATED the radioactivity measured after cells were lysed with NaOH (internalized). The total cell bound (=Surface + Internalized) radioactivity, with the statistical difference in comparison to IONs-SH (LNCaP: **A3,A4**, PC3: **B3,B4**), where (ns) *p* > 0.05, (*) *p* ≤ 0.05, (**) *p* ≤ 0.01, (***) *p* ≤ 0.001, (****) *p* ≤ 0.0001.

The cell binding studies included the IONs after the MPTES functionalization (IONs-SH, negative control), the IONs after coupling the PSMA (IONs-PSMA), the BN (IONs-BN) pharmacophore, or both (IONs-PSMA/BN). All the above IONs were directly labeled using anhydrous SnCl_2_ as the reducing agent of Na[^99m^Tc]TcO_4_^−^ and were used for the cell assays without further purification (provided RCC was above 94%) in PC3 and LNCaP cells. In order to investigate the internalization of nanoparticles in the cells, we tested them at 37°C and at 4°C, where internalization, as an energy-dependent process, is minimized. The results are summarized in Figure 8, while the added radioactivity per nanoparticle concentration expressed in cpm can be found in Supplementary Tables S1, S2, while the statistical analysis of results is presented in Supplementary Table S3 (supporting information). The top graphs in Figure 8 describe the surface bound (ACID) and the internalized radioactivity of each ^99m^Tc-labeled IONs species (LNCaP Figures 8A1,A2 and PC3 Figures 8B1,B2) in the two temperatures studied, while the bottom graphs show the total (surface + internalized) cell bound radioactivity and the statistical significance of each measurement in comparison to the controls (IONs-SH). An additional negative control with chemical blocking was not utilized because, as we have previously demonstrated (37), the chemical blocking experiments in concentrations (×4,000 excess) described in the literature (53) were not effective, this was possibly due to the multimerization of the pharmacophores on the surface of IONs. Further studies (beyond the scope of this work) are needed in order to optimize cell assays for such activated nanostructures.

In the case of the LNCaP cells, internalization (lysate) increased with the increase of the concentration at both 4°C and 37°C, while the saturation reaches a plateau. This phenomenon was observed for all cases of IONs, whether these were the controls (IONs-SH), the mono functionalized (IONs-PSMA), or the dual functionalized (IONs-PSMA-BN). However, the internalized fractions of the functionalized IONs were always significantly lower than the controls at 4°C, while the opposite phenomenon was observed at 37°C, where the internalized fractions (lysate) for IONs-PSMA and IONs-PSMA/BN were higher than the controls, even at the lowest concentrations studied. The total cell bound radioactivity was always higher for the functionalized IONs for concentrations above 2 mg/ml, at 37°C, while the opposite phenomenon was observed at 4°C, due to the minimization of specific internalization (Figures 8A3,A4).

Regarding PC3 cells, at 4°C the IONs-SH (control) showed an increase in surface binding (acid wash), which followed the increase of the concentration. The same trend was observed at 37°C. However, in the second case the amount of internalized IONs-SH (lysate) was much higher than the surface bound. The surface bound fraction of radioactivity was always higher for the IONs-BN compared to the internalized fraction, at both temperatures studied, indicating that the BN pharmacophore has changed their internalization process and that the IONs-BN have the same behavior as the BN antagonist. The BN antagonists are known for showing minimal internalization and for being mainly surface bound on PC3 cells (36, 54, 55). Moreover, in most cases, the internalized radioactivity reached a plateau at the highest concentrations studied (4 and 5 μg/ml), indicating a saturation in the process. For the dually functionalized IONs-PSMA/BN, results follow the same trends as IONs-BN, with the counts of the surface bound always being much higher than the internalized, at both temperatures studied. The total cell bound radioactivity in PC3 cells followed the same trend as the LNCaP cells, with the functionalized IONs showing higher values than the control at 37°C for concentrations above 1 mg/ml, and the opposite phenomenon happening at 4°C, where all energy depended processes are minimized.

Overall, in PC3 cells the functionalization of IONs with BN and PSMA/BN increased the surface and total binding at 37°C, while in LNCaP cells the functionalization with PSMA and PSMA/BN increased the internalized fraction and total binding, in comparison to the controls IONs-SH. These results are consistent with previous studies of our group on IONs functionalized with APTES and then with both pharmacophores, which were also tested at 4°C and 37°C with PC3 and LNCaP cancer cell lines (37).

## Conclusion

Herein, we have demonstrated how the molecular targeting of PSMA and GRPr in combination with nanotechnology can be utilized to enhance the properties of IONs as diagnostic probes for SPECT/MRI and PET/MRI hybrid imaging. To our knowledge, there are no probes with such characteristics in clinical practice or under investigational studies in humans. The radiolabeled IONs presented in this study carried either one or both pharmacophores, a Glu-CO-Lys ligand, targeting PSMA and a bombesin peptide, targeting GRPr, for dual targeting of prostate cancer. Radiolabeling with ^99m^Tc was accomplished with high efficiency, and the radiolabeled IONs were stable in the presence of serum, histidine, and DTPA. Moreover, IONs showed low toxicity levels in PC3 and LNCaP cancer cell lines in MTT studies and high biocompatibility in RBCs. Wound healing was affected after modification of IONs with the pharmacophores, providing further proof of concept for their activity, even after binding on IONs. The results were in accordance with known biological activities for the pharmacophores and with PSMA and BN receptor cell expression, i.e., the IONs carrying the BN antagonist presented anti-proliferating activity for PC3 cells, which express the GRP receptors. An analogous behavior was observed for the functionalized ^99m^Tc-IONs, i.e., IONs showed higher cell binding (at 37°C) for PC3 and LNCaP cells, after their modification with either one (^99m^Tc-IONs-PSMA, ^99m^Tc-IONs-BN) or both pharmacophores (^99m^Tc-IONs-PSMA/BN), in comparison to the controls (^99m^Tc-IONs-SH), providing *in vitro* proof of concept of our initial hypothesis.

IONs modification methods and *in vitro* results, presented in this study, open a vast range of possibilities for further surface modifications on the surface of IONs using various biocompatible, bioactive materials, ligands, and antibodies. The encouraging results of this research prompt us to continue with future *in vivo* studies in tumor models, in order to assess tumor targeting efficacy and dosimetry for imaging with SPECT/MRI and PET/MRI.

## Data Availability

The raw data supporting the conclusions of this article will be made available by the authors, without undue reservation.
