# Supplementary material for: Radiolabeled iron oxide nanoparticles functionalized with PSMA/BN ligands for dual-targeting of prostate cancer
Source: Front Nucl Med. 2023 Sep 20;3:1184309. doi: 10.3389/fnume.2023.1184309 (PMC11460297; doi:10.3389/fnume.2023.1184309)
Supplement: Supplementary file 1 [file Table1.docx]

**
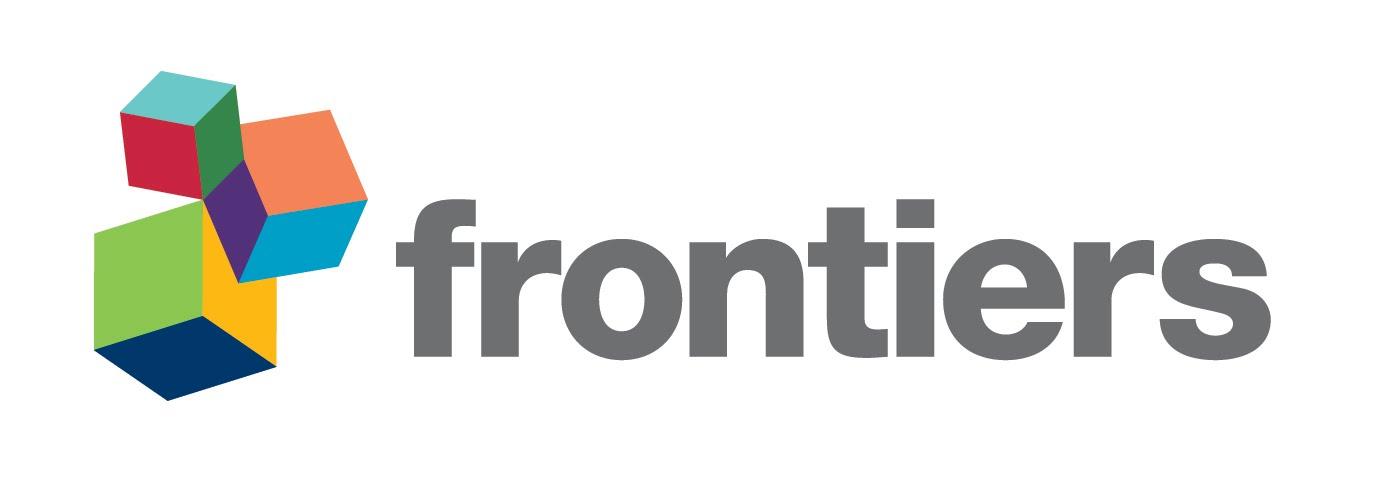
**

**Supporting information for**

**Radiolabeled Iron Oxide Nanoparticles Functionalized with PSMA/ΒΝ Ligands for Dual-Targeting of Prostate Cancer**

Danae Efremia Bajwa, Evangelia-Alexandra Salvanou, Maria Theodosiou, Theodora S. Koutsikou, Eleni Κ. Efthimiadou, Penelope Bouziotis, Christos Liolios

**Contents of SI**

Table S1 Initial radioactivity (cpm) added from each sample into LNCaP cancer cells (internalization study)...p.1

Table S2 Initial radioactivity (cpm) added from each sample into PC3 cancer cells (saturation study)………...p.2

Table S3. Statistical analysis of *in vitro* cell assays in LNCaP and PC-3 cells for total cell binding of the directly radiolabeled IONs ……………………………………………………………………………………………….p.3

**Table S1. Initial radioactivity (cpm) added from each sample into LNCaP cancer cells (internalization study).**

| **LNCaP** | | | |
| --- | --- | --- | --- |
| **IONs** | **C (μg/mL)** | **cpm at 37 ⁰C** | **cpm at 4 ⁰C** |
| [^99m^Tc]Tc-IONs-SH | 0.5 | 29408 | 19736 |
|  | 1 | 67973 | 38266 |
|  | 2 | 132024 | 70703 |
|  | 3 | 193867 | 100544 |
|  | 4 | 261960 | 127544 |
|  | 5 | 329963 | 157303 |
| [^99m^Tc]Tc-IONs-PSMA | 0.5 | 23311 | 2484 |
|  | 1 | 54391 | 23725 |
|  | 2 | 147579 | 56092 |
|  | 3 | 219366 | 84979 |
|  | 4 | 283451 | 117187 |
|  | 5 | 349210 | 149041 |
| [^99m^Tc]Tc-IONs-PSMA/BN | 0.5 | 21789 | 12527 |
|  | 1 | 65136 | 27433 |
|  | 2 | 138601 | 59784 |
|  | 3 | 208078 | 95969 |
|  | 4 | 275506 | 128237 |
|  | 5 | 342858 | 167172 |

**Table S2. Initial radioactivity (cpm) added from each sample into PC3 cancer cells (saturation study).**

| **PC3** | | | |
| --- | --- | --- | --- |
| **IONs** | **C (μg/mL)** | **cpm at 37 ⁰C** | **cpm at 4 ⁰C** |
| [^99m^Tc]Tc-IONs-SH | 0.5 | 43360 | 37182 |
|  | 1 | 94091 | 77966 |
|  | 2 | 191515 | 165639 |
|  | 3 | 291531 | 264918 |
|  | 4 | 391151 | 359448 |
|  | 5 | 481987 | 460128 |
| [^99m^Tc]Tc-IONs-BN | 0.5 | 49338 | 21141 |
|  | 1 | 118148 | 52134 |
|  | 2 | 252659 | 145628 |
|  | 3 | 376214 | 239834 |
|  | 4 | 508546 | 357676 |
|  | 5 | 615277 | 465048 |
| [^99m^Tc]Tc-IONs-PSMA/BN | 0.5 | 33254 | 35074 |
|  | 1 | 117113 | 71575 |
|  | 2 | 252342 | 156660 |
|  | 3 | 378383 | 269858 |
|  | 4 | 514739 | 367250 |
|  | 5 | 678700 | 464280 |

**Table S3. Statistical analysis^$^ of *in vitro* cell assays in LNCaP and PC-3 cells for total cell binding of the directly radiolabeled IONs**

| PC3 | | | | | | | | | | | | | | | | |
| --- | --- | --- | --- | --- | --- | --- | --- | --- | --- | --- | --- | --- | --- | --- | --- | --- |
| 37 ^o^C | | | | | | | | | 4 ^o^C | | | | | | | |
|  | IONs-SH | | IONs-BN | | | IONs-PSMA/BN | | | IONs-SH | | IONs-BN | | | IONs-PSMA/BN | | |
| C (μg/mL) | cpm | SD | cpm | SD | P | cpm | SD | P | cpm | SD | cpm | SD | P | cpm | SD | P |
| 0.5 | 591 | 133 | 1164 | 372 | ns | 732 | 123 | ns | 1620 | 305 | 447 | 56 | ** | 109 | 16 | *** |
| 1 | 1066 | 131 | 2216 | 831 | * | 2586 | 971 | ** | 2184 | 319 | 975 | 210 | ** | 272 | 53 | **** |
| 2 | 2070 | 325 | 5563 | 409 | **** | 2942 | 438 | * | 3275 | 1140 | 1563 | 251 | **** | 449 | 165 | **** |
| 3 | 2819 | 202 | 6225 | 893 | *** | 4617 | 1147 | *** | 2699 | 351 | 1833 | 208 | * | 703 | 84 | **** |
| 4 | 4399 | 605 | 7282 | 918 | **** | 5430 | 472 | * | 3577 | 1086 | 2256 | 426 | *** | 729 | 118 | **** |
| 5 | 5513 | 471 | 8472 | 714 | **** | 6492 | 665 | * | 6981 | 1025 | 3160 | 562 | **** | 953 | 93 | **** |
| LNCaP | | | | | | | | | | | | | | | | |
| 37 ^o^C | | | | | | | | | 4 ^o^C | | | | | | | |
|  | IONs-SH | | IONs-PSMA | | | IONs-PSMA/BN | | | IONs-SH | | IONs-PSMA | | | IONs-PSMA/BN | | |
| C (μg/mL) | cpm | SD | cpm | SD | P | cpm | SD | P | cpm | SD | cpm | SD | P | cpm | SD | P |
| 0.5 | 1533 | 246 | 2299 | 306 | ns | 1870 | 261 | ns | 1704 | 204 | 372 | 30 | * | 794 | 172 | ns |
| 1 | 2553 | 172 | 3646 | 650 | ns | 4332 | 162 | ns | 3261 | 464 | 1347 | 250 | ** | 1923 | 203 | * |
| 2 | 6605 | 1126 | 8979 | 1383 | * | 9345 | 1152 | * | 5674 | 823 | 2493 | 164 | **** | 3654 | 273 | ** |
| 3 | 11039 | 1168 | 14444 | 385 | ** | 13329 | 2574 | * | 9580 | 884 | 3477 | 500 | **** | 5586 | 271 | **** |
| 4 | 12694 | 1509 | 18679 | 1687 | **** | 20154 | 2365 | **** | 16879 | 2216 | 6310 | 1409 | **** | 8205 | 778 | **** |
| 5 | 14411 | 794 | 20273 | 1283 | **** | 20935 | 552 | **** | 20853 | 869 | 7715 | 730 | **** | 11755 | 588 | **** |

**^$^ Statistical Analysis**

Statistical analysis was conducted with Graph Pad Prism using the ordinary Two-way ANOVA tests (Dunnett’s multiple comparisons test), alpha = 0.05, where (ns) P > 0.05, (*) P ≤ 0.05, (**) P ≤ 0.01, (***) P ≤ 0.001, (****) P ≤ 0.0001.
